# The Effects of Individual Differences, Non-Stationarity, and the Importance of Data Partitioning Decisions for Training and Testing of EEG Cross-Participant Models

**DOI:** 10.3390/s21093225

**Published:** 2021-05-06

**Authors:** Alexander Kamrud, Brett Borghetti, Christine Schubert Kabban

**Affiliations:** Department of Electrical and Computer Engineering, Air Force Institute of Technology, Wright-Patterson AFB, OH 45433, USA; brett.borghetti@afit.edu (B.B.); christine.schubert@afit.edu (C.S.K.)

**Keywords:** EEG, deep learning, non-stationarity, individual differences, inter-subject variability, covariate shift, cross-participant, inter-participant

## Abstract

EEG-based deep learning models have trended toward models that are designed to perform classification on any individual (cross-participant models). However, because EEG varies across participants due to non-stationarity and individual differences, certain guidelines must be followed for partitioning data into training, validation, and testing sets, in order for cross-participant models to avoid overestimation of model accuracy. Despite this necessity, the majority of EEG-based cross-participant models have not adopted such guidelines. Furthermore, some data repositories may unwittingly contribute to the problem by providing partitioned test and non-test datasets for reasons such as competition support. In this study, we demonstrate how improper dataset partitioning and the resulting improper training, validation, and testing of a cross-participant model leads to overestimated model accuracy. We demonstrate this mathematically, and empirically, using five publicly available datasets. To build the cross-participant models for these datasets, we replicate published results and demonstrate how the model accuracies are significantly reduced when proper EEG cross-participant model guidelines are followed. Our empirical results show that by not following these guidelines, error rates of cross-participant models can be underestimated between 35% and 3900%. This misrepresentation of model performance for the general population potentially slows scientific progress toward truly high-performing classification models.

## 1. Introduction

EEG analysis has been a useful tool in neuroscience for decades in both clinical settings and the medical research community, proving to be useful for numerous applications such as classifying sleep patterns, epilepsy, identifying patterns of attention deficit hyperactivity disorder (ADHD), levels of mental workload [1,2], and emotion recognition [3]. EEG has also been useful for neural engineering with Brain–Machine Interfaces (BMIs), primarily due to EEG being used in combination with machine learning. Over the past decade, deep learning (DL) has been increasingly used to improve performance within models, allowing for automatic end-to-end processing and classification of the data, to include feature extraction using sequence models. Despite these improvements in model selection, the challenges of EEG’s non-stationarity and inter-participant variability are still present [4,5] pp. 499–502.

One of the most significant challenges in building EEG classification models that are intended for use on any individual’s EEG (cross-participant model) is accounting for the covariate shift that occurs due to EEG’s non-stationarity and inter-participant variability [6,7,8,9,10]. Covariate shift in machine learning is a difference in the input distributions of the training and testing datasets [11]. This difference can significantly affect model performance, as a general guideline and assumption that is used in supervised machine learning is that these two input distributions are independent and identically distributed (i.i.d. assumption). Without this assumption, many theoretical guarantees and bounds on minimizing the test error are lost. For EEG cross-participant classification models, this covariate shift and its effects will always be present when the model classifies EEG data belonging to a participant that the model has not seen. However, models should be tested with data, which is representative of the data they will predict upon in the real world, and thus, EEG cross-participant models should be tested with unseen participants. Therefore, as a best practice in reporting accurate model performance for models intended to classify any individual’s EEG, EEG cross-participant models should always be validated and tested using EEG data that comes from participants the model has not trained upon.

Despite previous work showing that EEG has inter-participant variability [5] pp. 499–502, and that this inter-participant variability leads to covariate shift when EEG models are tested with an unseen participant [6,7,8,9,10], the majority of EEG studies built to classify any individual’s EEG do not follow this best practice of testing the model with unseen participants. In a recent literature review of deep learning-based EEG models by Roy et al., only 23 out of 108 cross-participant models utilized some method of proper dataset partitioning to ensure the model was tested with a participant that was not used for training [3]. This same literature review also compared the number of studies exploring models built for a specific individual (within-participant) versus cross-participant, and they found that since 2016, the growing trend has shifted toward building cross-participant models, with the latest ratio of studies researching cross-participant models to within-participant models being over 5:1 [3]. With this ever-growing popularity in EEG cross-participant models, it is critical that the body of research corrects its trend by properly using EEG data from unseen participants for validation and testing. By not following this best practice, the research pool may become increasingly diluted with studies reporting model performance metrics that are unrealistic and unrepresentative of the model’s true ability. Additionally, data repositories that split data into training and testing datasets prior to being made available for download, such as Kaggle [12] and the University of California, Irvine (UCI) machine learning data repository [13], should also take this best practice into account. In this paper, we aim to present to the reader the importance of proper dataset partitioning.

This paper has the following structure. First, in Section 2, a well-established background is presented to ground the reader in regard to covariate shift and inter-participant variability within EEG; then, we fully articulate the problem of improper dataset partitioning using this background knowledge. Next, in Section 3, we demonstrate the effects of covariate shift and inter-participant variability both mathematically and in simple models, presenting evidence for the effects of these phenomena at a fundamental level. Finally, in Section 4, we utilize five publicly available datasets to present empirically the difference in model performance when following and not following this best practice of proper model validation and testing. We close with discussion in Section 5 and conclusions and future work in Section 6.

## 2. Background

### 2.1. Covariate Shift

For supervised machine learning, a standard guideline is that the training input distribution *P_TR_*(*x*) is equivalent to the test input distribution *P_TE_*(*x*) [11]. However, when these two distributions are not equivalent *P_TR_*(*x*) ≠ *P_TE_*(*x*), then there is typically a decrease in performance for most machine learning models. This form of dataset shift is referred to as covariate shift. This can happen for a number of reasons, such as the training and testing data being drawn from different populations, a lack of randomness in the number of trials/observations, an inadequate amount of them, or other biased sampling measures; in the case of EEG, covariate shift is due to individual differences and non-stationarity [10,14]. 

**Figure 1:**
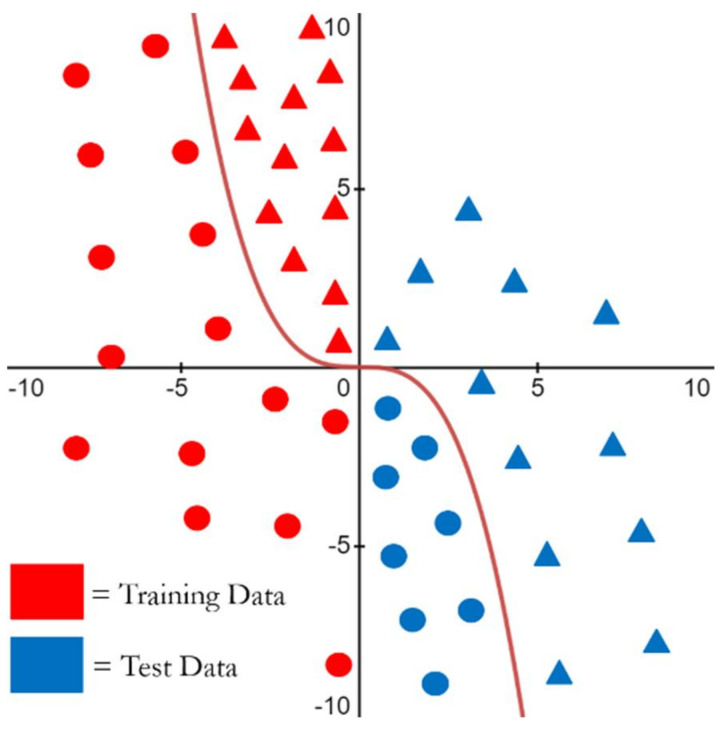
Simple example of covariate shift in classification data. Two classes of data are represented by circles and triangles, with the training dataset marked in red and the test dataset marked in blue. The true decision boundary between the two classes follows the function $y=-x^{3}$.

Below, in Figure 1, we see a simple example of covariate shift. Here, there is a classification boundary between two different classes, one represented by circles, and the other represented by triangles, with the classification boundary following the function $y=-x^{3}$. The training dataset is marked in red and the test dataset is marked in blue. If we train a machine learning algorithm on only the training dataset and then test it on similar data such that *P_TR_*(*x*) = *P_TE_*(*x*), then the model will be able to perform very well when tested, since the classification boundary is well defined between the two classes. In fact, many functions could easily define a reasonable boundary in this case; for example, $y=x^{2}/3$ or *y* = 2|*x*| would yield good performance at discriminating the two classes of the training set shown in Figure 1. However, if we trained the model using only the red training data and tested with the blue testing data, the machine learning algorithm would have been trained with different data than it would be tested with (*P_TR_*(*x*) ≠ *P_TE_*(*x*)), and it is unlikely that during training, the machine learning algorithm would have been able to discover the more complicated underlying discriminator function $y=-x^{3}$ having used only the red training data. Thus, the model trained only on the training data would perform poorly for classification of the test data, because the data distribution of the features from the training data and the distribution of the features from the test data are different.

There are a number of different methods that can be used to detect if covariate shift is present due to the input distributions from two datasets being different. Given two datasets, $P_{TR}\left(x\right)\;\mathrm{and}\;P_{TE}\left(x\right)$, one method is to calculate *how* different the two probability distributions of the two datasets are,

$$\begin{array}{c} D_{KL}(P_{TR}||P_{TE})=E_{x\sim P_{TR}}\left[log\frac{P_{TE}\left(x\right)}{P_{TR}\left(x\right)}\right]=E_{x\sim P_{TR}}[\log\left(P_{TR}\left(x\right)\right)-\\ \log\left(P_{TE}\left(x\right)\right)]. \end{array}$$

Another method for covariate shift detection is through visualization of the distributions in low-dimensional space using dimensionality reduction techniques. Manifold learning techniques such as t-Distributed Stochastic Neighbor Embedding (t-SNE), multi-dimensional scaling (MDS), IsoMap, and others, are useful for this as they capture non-linear information in the data [15] pp. 209–226. t-SNE is an unsupervised machine learning algorithm that is widely used for data visualization as it is particularly sensitive to local structure and reduces the tendency to crowd points toward the center of low-dimensional space [16]. As an unsupervised machine learning algorithm, t-SNE does not use labels of data for its learning, and it solely uses the features of each observation to perform its algorithm. It does this by first constructing a probability distribution for all pairs of observations in high-dimensional space such that similar observations (observations that are closer to one another in feature space) are assigned a higher probability of being neighbors, and dissimilar observations (observations that are further apart in feature space) are assigned a lower probability of being neighbors. Then, a new dataset is created with the same number of observations, but it is now spread randomly in low-dimensional feature space. It uses a Student’s *t*-distribution to compute the similarity between all pairs of observations in low-dimensional space to create a second probability distribution and then uses gradient descent to iteratively shift the observations such that the KL divergence between the two different distributions is minimized. The main limitations of t-SNE are that it is computationally expensive and that the algorithm uses a non-convex objective function (KL divergence minimized using gradient descent, but initiated randomly), meaning multiple executions of the algorithm can lead to different embeddings (mappings of high-dimensional space to low-dimensional space). The dimensions of t-SNE are also difficult to interpret, as they are arbitrary distances that represent that closer neighboring points in low-dimensional space are likely to be neighbors in high-dimensional space [17].

**Figure 2:**
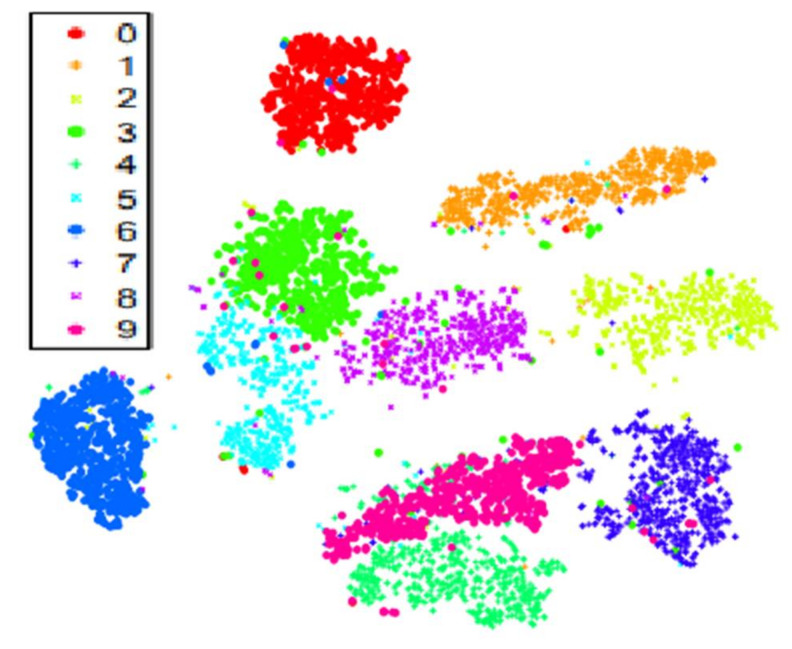
Example of 2D visualization using t-SNE on the MNIST dataset [16,18]. The dimensions of t-SNE are arbitrary distances that represent that closer neighboring points in low-dimensional space are likely to be neighbors in high-dimensional space.

Figure 2 shows an example of previous work utilizing t-SNE for high-dimensional data visualization outside of the EEG domain, with t-SNE performed on the well-known MNIST dataset, with the clusters corresponding to different input distributions within the data, and the colors corresponding to different classes [16,18]. 

t-SNE can also be used to visually detect covariate shift. A common example of covariate shift is when the testing data is partitioned from a subset of the clusters (i.e., participants for EEG), and the training data is partitioned from a different and separate subset of clusters; e.g., if in Figure 2 class 0 (red) was selected as the test data and classes 1–9 were selected as the training data. Cluster analysis algorithms such as k-means clustering or fuzzy c-means clustering can be utilized to identify if the training and testing data belong to separate clusters [19]; however, a simpler method to detect this is through visual inspection of the t-SNE graph. One can separately label the training and test data in the graph (e.g., with different colors) and then visually inspect to see if the training and test data correspond to separate clusters within the graph (covariate shift). Visual inspection for clusters involves identifying that for the majority of observations in one class, the majority of the nearest neighbors for those observations also belong to the same class, with a clear boundary between its class (cluster) and another class, meaning there is little to no overlap. This simple method of visual inspection also provides the benefit of visualizing the high-dimensional data in 2D.

### 2.2. Non-Stationarity and Individual Differences

One of the significant challenges associated with EEG analysis and classification is that EEG is both non-stationary [4] and that there are individual differences in EEG signals across individuals that result in inter-participant variability [5] pp. 499–502. EEG non-stationarity is due to a variety of internal and external causes, such as brain activity causing continual changes in states of neuronal assemblies [20], user attention levels, user fatigue, sensor equipment used, and scalp placement of electrodes [21]. Similar to non-stationarity, the individual differences in EEG signals are also due to a variety of factors, such as differences in variability in frequency peaks for individuals due to differences in personality traits [22], genetic variations [23,24,25], gamma–aminobutyric acid concentrations in the brain [26,27], and memory task performance [28]. 

These individual differences are underlying shifting covariates across participants, and they result in a change in the input distributions across all participants, while the conditional distribution of the output class *y* given the input feature vector ***x*** stays the same, resulting in a covariate shift for cross-participant machine learning models when they are tested upon EEG from participants that the model has not seen [6,7]. Thus, because of this inherent inter-participant variability in EEG signals, different strategies need to be used when performing EEG analysis [5] pp. 499–502 and training of cross-participant models [29].

### 2.3. Approaches to Data and Problem Formulation

When developing an EEG classification model, it is likely that it will belong to one of two main types of EEG models, either within-participant (a.k.a. intra-subject) or cross-participant (a.k.a. inter-subject) [3]. A within-participant model is one that intends to perform accurate classification of EEG for one individual and is thus built using only data from one participant. A cross-participant model is one that intends to perform classification on multiple individuals and is thus built using data from multiple participants. By training on data from multiple individuals, the goal is that the model becomes invariant to inter-participant variability, learning a function that accurately maps EEG input to the desired output label for most people. Additionally, cross-participant models can be built for different purposes and goals, such as for specific populations or for the general population. For example, the goal of a cross-participant BMI model could be to perform classification on only those specific individuals that use that specific BMI machinery. However, a more typical cross-participant model is one in which results are reported as though they are indicative of the model’s ability to perform classification on the general population and thus any individual. 

**Figure 3:**
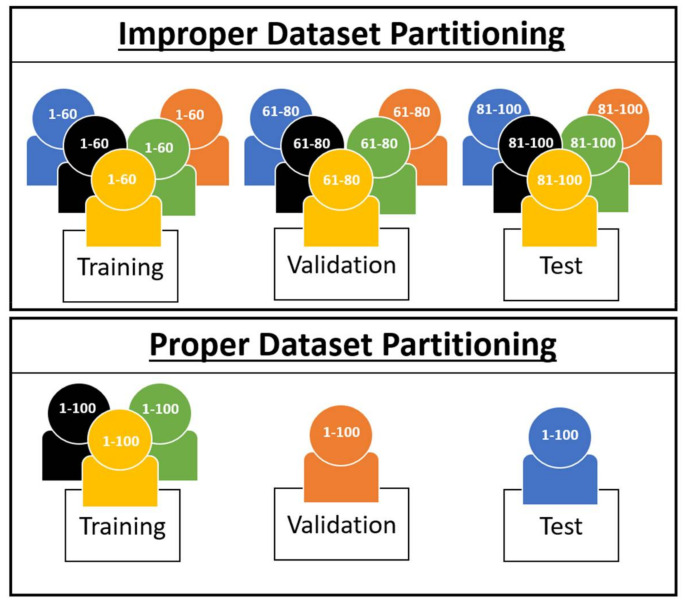
Two examples of creating the training, validation, and testing datasets with data from five participants. Numbers correspond to unique observations within each participant’s dataset, with “1–60” referring to observations #1 through #60, “61–80” referring to observations #61 through #80, etc. The top illustrates improper dataset partitioning: data from each participant are used for all three datasets. In the top panel, while no unique observation is in more than one subset, each participants’ data is still present in each subset. The bottom illustrates proper dataset partitioning: each participant’s data are present in no more than one of the subsets.

Each of these model types require different approaches to data partitioning across participants in order to report results that are accurate for their intended goal and target population. The within-participant model is more straightforward, as there is only one participant for both training, validation, and testing. However in cross-participant models, there are data from multiple participants, and because of the inter-participant variability that is inherent in EEG from individual to individual, how participants are used in cross-participant models for training, validation, and testing can have significant effects on model performance due to the differences in input distributions from individual to individual [6,7]. For example, if a cross-participant model is tested using data from an unseen participant, then the model’s classification performance will be reduced due to the resulting covariate shift of this individual’s unseen data. If a cross-participant model is only intended to perform classification on the same population that it is training upon and not also unseen individuals, as is in some BMI models, then ensuring the model is tested with unseen individuals is not necessary. However, for cross-participant models in which the model is intended for the general population and therefore unseen individuals, data should be prepared such that *participants* that are used for training are not also used for validation or testing, and *participants* used for validation are not also used for testing; otherwise, the model’s performance will not accurately reflect its intended purpose of classification upon unseen individuals. This means that if participant A is used for training, then not even a single observation from participant A should be used for validation or testing, and if participant B is used for validation, then not even a single observation from participant B should be used for testing. An example of this method of proper vs. improper dataset partitioning for general population cross-participant models is depicted in Figure 3. It is also worth noting that proper validation of general population cross-participant models does not exclude the use of cross-validation (CV) as a performance evaluation technique. Instead, CV merely needs to be modified so that for each fold, *participants* used in training are not also used for validation, such as a Leave-One-Participant-Out approach or a Leave-N-Participants-Out approach.

Cross-participant models have significantly grown in popularity in recent years [3]; however, the majority of studies using cross-participant models do not follow this proper method of dataset partitioning. In Roy et al.’s literature review of deep learning-based EEG models, out of 108 studies using cross-participant models, only 23 utilized some method of proper dataset partitioning with a Leave-N-Participants-Out approach or a Leave-One-Participant-Out approach [3]. This results in the majority of studies having overestimated performance metrics—suggesting readers use models which, when used in scenarios involving the general population, may not perform as well as they were reported to have performed in the research. To obtain meaningful estimates of performance in the general population, cross-participant models need to follow proper dataset partitioning, as shown in Figure 3. Alternately, if the intent is not to use the model in the general population and is instead a tailored model designed for a specific population subset, the study should specifically state that the model’s intended goal is only to perform classification upon the individuals it has been trained upon, to prevent the reader from incorrectly believing its efficacy would be similar in the general population. 

In the following sections, we demonstrate in greater detail how covariate shift occurs, as well as its effects, in both simple model examples (Section 3), and in real-world, publicly available datasets (Section 4). 

## 3. Initial Demonstration

To build understanding for how covariate shift manifests in any data, we utilize an initial demonstration of its effects in three settings: (1) first, we define covariate shift mathematically and illustrate how its effects on the expected loss of the test distribution can be accounted for; (2) next, we depict covariate shift using t-SNE, specifically using EEG data; and (3) finally, we demonstrate how we can affect covariate shift in EEG data by reducing the inter-participant variability through data transformations, thus increasing the model accuracy of properly validated EEG cross-participant models. 

### 3.1. Defining and Estimating the Effects of Covariate Shift

In order to understand covariate shift at its fundamental level, we first define supervised learning. Supervised learning is the task of learning a function ƒ(**x**), which maps an input vector ***x*** to a labeled output *y*, typically done by estimating the conditional probability *p*(***y***|***x***) [30] pp. 102–104. In order to estimate this function ƒ(***x***), a loss function ℓ(ƒ(***x***),***y***) provides a measure of the difference between the true output *y* and the estimated $\hat{y}$ for the input vector ***x***, with the loss function producing smaller values if $\hat{y}$ is correct and larger values if $\hat{y}$ is incorrect. Thus, the task of learning involves minimizing the expected loss of ℓ(ƒ(***x***),***y***) over the probability density *p*(***x***,***y***|***λ***) (parameterized by ***λ***), i.e., minimizing the loss ℓ(ƒ(***x***),***y***) over all possible inputs ***x*** [31],

(1) $$E_{\left(x,y\right)\sim p(x,y|\lambda )}\left[l\left(ƒ\left(x\right),y\right)\right]=\iint l\left(ƒ\left(x\right),y\right)p\left(x,y|\lambda \right)dxdy.$$

However, in practice, the distribution *p*(***x***,***y***|***λ***) is unknown and thus replaced by the empirical distribution, which can be estimated from training samples. If there is the set of samples *L* drawn from *p*(***x***,***y***|***λ***), then Equation (1) becomes the objective of minimizing the empirical loss [31],

(2) $$E_{\left(x,y\right)\sim L}\left[l\left(ƒ\left(x\right),y\right)\right]=\frac{1}{\left|L\right|}\underset{\left(x,y\right)\in L}{\sum }l\left(ƒ\left(x\right),y\right).$$

After minimizing the empirical loss and a prediction model is learned, the model is tested with the set of test samples *T* drawn from *p*(***x***,***y***|***λ***), where *T* does not contain any samples from *L* that were used to minimize the empirical loss.

If the training data and testing data are independently and identically distributed (i.i.d.), meaning that every single observation of training and testing data are sampled independently and from the same distribution of *p*(***x***,***y***|***λ***), then we expect that minimizing the expected training loss will in general also minimize the expected test loss [31]. This is an assumption that is common for many predictive models and is referred to as the i.i.d. assumption. However, many models are developed under conditions such as non-stationary signals or covariate shift. In these conditions, the i.i.d. assumption no longer holds, as the training and testing data come from different distributions, e.g., *p*(***x***,***y***|***λ***) (parameterized by ***λ***) for the training data, and *p*(***x***,***y***|θ) (parameterized by θ) for the testing data. As we no longer have the assumption of i.i.d. data, then we can no longer expect that minimizing the expected training loss also in general minimizes the expected test loss,

(3) $$\begin{array}{c} argmin\\ ƒ \end{array}E_{\left(x,y\right)\sim p(x,y|\lambda )}\left[l\left(ƒ\left(x\right),y\right)\right]\neq \begin{array}{c} argmin\\ ƒ \end{array}E_{\left(x,y\right)\sim p(x,y|\theta )}\left[l\left(ƒ\left(x\right),y\right)\right].$$

One method to address this lack of minimizing the expected test loss under covariate shift is through loss rescaling. Shimodaira proposed that if the training and test distributions are known, that the expected test loss could be minimized by appropriately weighting the training loss for each ***x*** with instance-specific weights $\frac{p(x|\theta )}{p(x|\lambda )}$ [31,32],

(4) $$E_{\left(x,y\right)\sim \theta }\left[l\left(ƒ\left(x\right),y\right)\right]=E_{\left(x,y\right)\sim \lambda }\left[\frac{p(x|\theta )}{p(x|\lambda )}l\left(ƒ\left(x\right),y\right)\right].$$

This loss rescaling results in larger loss values for instances of ***x*** where there are fewer training samples than test samples (weight ratio > 1), and smaller loss values for instances of ***x*** where there are more training samples than test samples (weight ratio < 1). Thus, in a dataset without covariate shift between training and test, more weight ratios’ magnitudes would be close to unity because the features of the training data have a similar distribution to the features of the test data. Conversely, in a dataset with covariate shift between training and test, fewer ratios would be closer to 1.0, and more weight ratios would have magnitudes differing further from 1.0 because the training distribution and test distribution differ in their feature distributions. 

While loss rescaling could be used to adjust machine learning performance outcomes, implementing loss rescaling can be difficult to achieve. As can be seen in Equation (4), for each instance of ***x*** with positive *p*(***x***,***y***|θ), there must also be a positive *p*(***x***,***y***|***λ***); otherwise, there is a zero denominator, meaning this loss rescaling can only occur if the training distribution covers the entire support of the test distribution [31]. In high-dimensional data, it is more difficult to have this coverage due to the curse of dimensionality, i.e., that the sparsity of the data increases exponentially as the number of dimensions (e.g., number of features) increase. High-dimensional data are common in EEG datasets due to the nature of recording brain activity with high numbers of channels (i.e., scalp electrodes), and additionally, if spectral features are utilized, there are multiple frequency bands that could be extracted for each channel; it is not uncommon to collect spectral energy from five frequency bands across 64 electrodes for a total of 320 features in ***x***. 

While loss rescaling is unlikely to be useful for determining better estimates of performance in real-world EEG machine learning models, it can be useful for exploring effects of covariate shift in low-dimensional spaces. Next, we present a low-dimensional transformation of EEG datasets using Principal Components Analysis (PCA) in order to explore the performance differences between improper and proper partitioning of datasets for machine learning models. 

We demonstrate the effects of these loss-rescaling weight ratio values $\frac{p(x|\theta )}{p(x|\lambda )}$ [31,32] using the spectral features of the Driver Fatigue dataset [33] described in Section 4.1. First, the input vectors are log transformed to reduce skew, and the dataset is partitioned into two separate training and test datasets using the proper and improper methods:

- For improper dataset partitioning, all participant data were shuffled together and one-twelfth of the data were randomly selected for the test set, with the remaining data selected for the training set.
- For proper dataset partitioning, one participant was selected for the test set, and the remaining 11 participants were selected for the training set.

**Figure 4:**
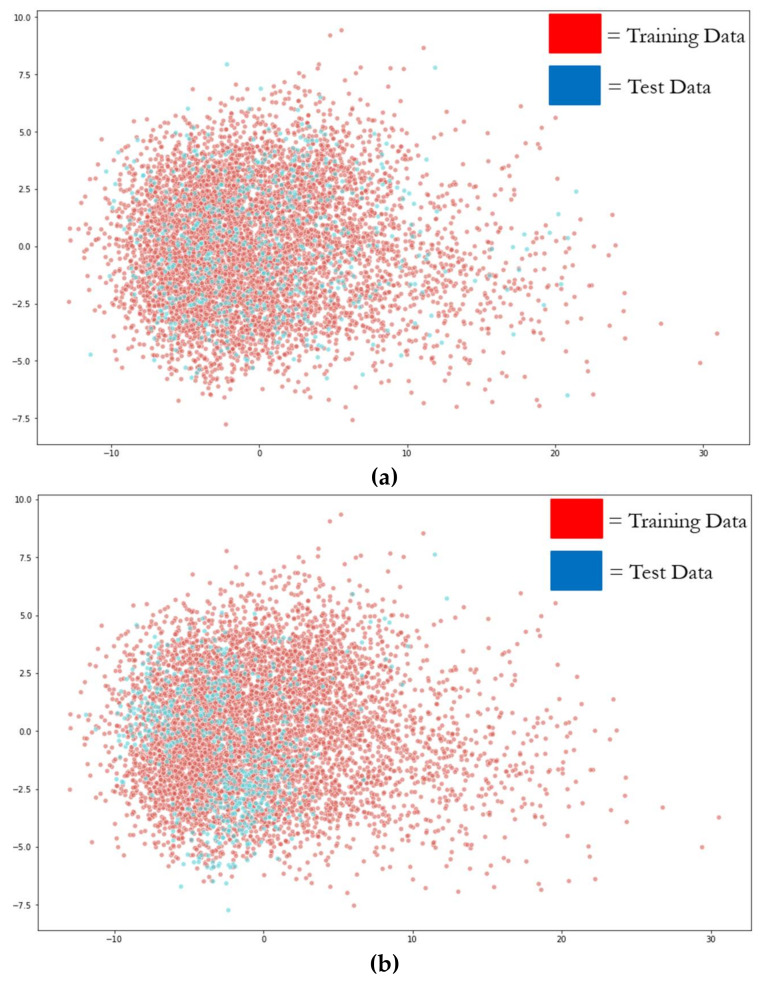
PCA projection of the first two principal components for (**a**) Improper and (**b**) Proper methods of dataset partitioning for spectral features of the Driver Fatigue dataset [33]. Red dots represent training data observations, and blue dots represent test data observations. Note that in the improper (**a**) that the test distribution is more uniformly spread throughout the training distribution, as all 12 participants are in the test distribution, while in the proper (**b**), the test distribution is more clustered due to the entire test distribution belonging to a single participant. These graphs are newly generated from the data obtained in the Driver Fatigue dataset [33].

Then, PCA was applied separately to the improper and proper datasets in order to reduce the dimensionality of the data to its first two principal components, with the amount of variance explained by the first two components being 0.72 for improper and 0.73 for proper. PCA dimensionality reduction is applied to both datasets so that the training distribution is more likely to cover the entire support of the test distribution [31]. Figure 4a,b depict the graphs for improper and proper dataset portioning: red dots representing training data observations, and blue dots representing test data observations. Note that in Figure 4a (improper), the test distribution is more uniformly spread throughout the training distribution, as all 12 participants are included in the test distribution, while in Figure 4b (proper), the test distribution is more clustered due to the entire test distribution belonging to a single participant.

Recall that the loss rescaling weight ratios represent a multiplier on the loss function in order to better estimate the expected real loss function from the loss estimate produced during evaluation of a model when there was a covariate difference between the test (*p*(***x***,***y***|θ)) and training sets (*p*(***x***,***y***|***λ***)) used for machine learning. Ratios with values higher than 1 imply that there are more test data than training in this region; thus, the importance of the loss value in this region needs to be magnified; conversely, in regions with ratios smaller than 1, there is less test data than training data, meaning the loss values in this region are less important and their contribution to overall performance should be suppressed. 

**Figure 5:**
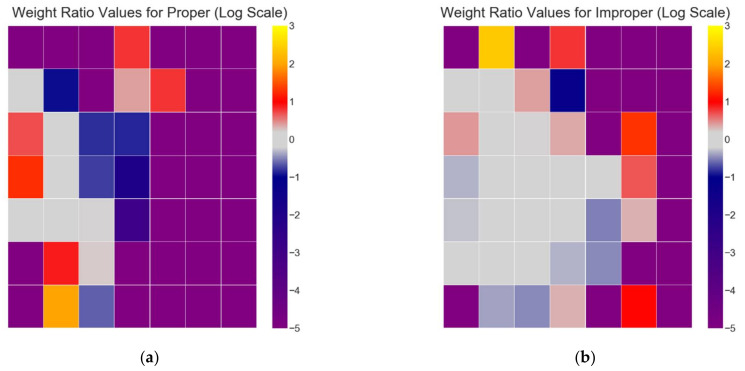

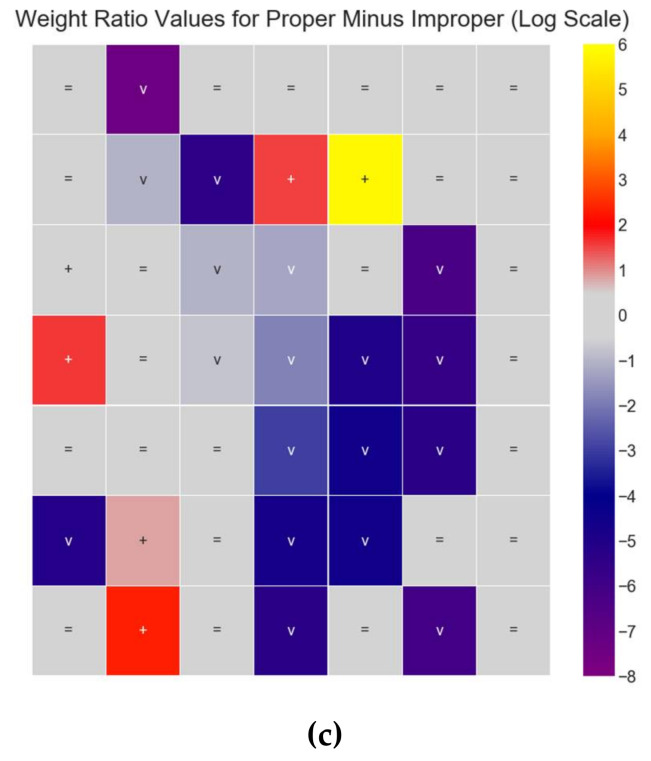
Heat maps for the log-transformed weight ratio values generated using two-dimensional histograms for (**a**) Proper (log(proper + ε)) and (**b**) Improper (log(improper + ε)) ($\varepsilon =1.0\times 10^{-5}$ ) methods of dataset partitioning for spectral features of the Driver Fatigue dataset [33]. Graph (**c**) depicts the difference in log-transformed weight ratio values between the proper and improper methods (log(proper + ε)—(log(improper + ε)), with labels for each bin indicating approximately equal weights (=), a significant negative delta (v), or a significant positive delta (+). These heat maps are newly generated from the data obtained in the Driver Fatigue dataset [33].

To calculate the loss rescaling weight ratio values $\frac{p(x|\theta )}{p(x|\lambda )}$ [31,32] within these datasets, some method of density estimation of the marginal input distributions is required; for the purposes of visualization and discussion, we utilize two-dimensional histogram estimators generated across a 7 × 7 grid of bins for each dataset (# of bins = 49). To help visualize this, imagine a 7 × 7 grid placed over the observations in each graph of Figure 4a and b, with the grid extending from the minimum values within the dataset, to the maximum values within the dataset, for both the X and Y axes. The number of training and testing observations within each histogram bin are calculated and normalized, providing our density estimation for the marginal input distributions, and subsequently the weight ratio values $\frac{p(x|\theta )}{p(x|\lambda )}$ for each bin. To better display the magnitude of difference in these weight ratio values, we display them in log scale, with a small value ($\varepsilon =1.0\times 10^{-5}$) added to the ratio values to avoid undefined values of log(0). This results in the log-transformed heat maps seen in Figure 5a–c, with Figure 5a being the log-transformed weight ratio values for the proper dataset $\left(\;\log\left(proper+\varepsilon \right)\right)$, Figure 5b being the log-transformed weight ratio values for the improper dataset $\left(\log\left(improper+\varepsilon \right)\right)$, and Figure 5c being the difference between the log transformed weight ratio values for proper minus improper $\left(\log\left(proper+\varepsilon \right)-\log\left(improper+\varepsilon \right)\right)$.

Figure 5a depicts that for the proper dataset partition, there are few bins (≈7) with a weight ratio close to 0, and many bins that are less than 0 (with many equal to −5, i.e., log(ε)) or greater than 0. In contrast, Figure 5b depicts that for the improper dataset partition, there are more bins (≈14) with a weight ratio close to 0, and fewer bins that are less than 0 or greater than 0. Bins that are less than 0 for proper are also darker blue than bins that are less than 0 for improper, indicating the training data have a more similar distribution to the test for improper vs. proper. In Figure 5c, the difference of the log-transformed weight ratio values between the two heat maps (proper minus improper) indicates that approximately half of the bins have a delta of 0, and the other half of the bins have a delta that is significantly less than 1.0 or significantly greater than 1.0. This signifies that there can be significant differences in the weights required to rescale the loss depending on how the data are partitioned, with significantly more loss rescaling being required for the proper method of dataset partitioning vs. the improper method. This significant difference in loss rescaling between the two methods is indicative of proper dataset partitioning resulting in a covariate shift, and because the only difference in partitioning between the two methods is how participants are distributed, it is also indicative of an unseen participant resulting in covariate shift.

### 3.2. Covariate Shift in EEG

In Section 2.1, we discussed how t-SNE can be utilized in order to detect covariate shift in data, and in Section 2.2, we discussed how covariate shift is inherent in EEG models due to the nature of EEG’s non-stationarity and the individual differences that result in inter-participant variability. Here, we utilize t-SNE to visually showcase why this inter-participant variability leads to the effect of covariate shift in EEG cross-participant models. As mentioned previously, t-SNE allows one to inspect for covariate shift in the data by first applying the unsupervised technique and then visually exploring the data in 2D space, examining it to see if the clusters of training data and testing data are isolated from one another through visual inspection.

**Figure 6:**
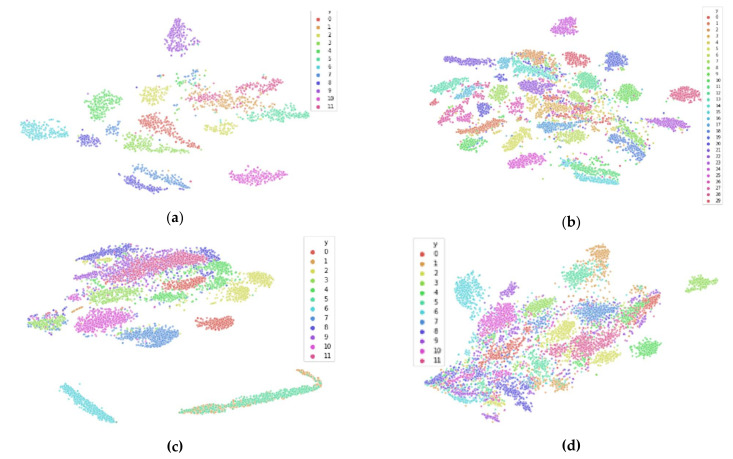
Example of using t-SNE for 2D dimensionality reduction and visualization of datasets utilized within this research, with colors corresponding to participants within the datasets, showcasing that inter-participant variability is present across different tasks and participant populations. Datasets depicted here are spectral features of the (**a**) PTSD, (**b**) Schizophrenia, (**c**) and Driver Fatigue datasets; and (**d**) Entropy features of the Driver Fatigue dataset. The dimensions of t-SNE are arbitrary distances that represent that closer neighboring points in low-dimensional space are likely to be neighbors in high-dimensional space.

We perform t-SNE on spectral features of the PTSD [34], Schizophrenia [35], and Driver Fatigue datasets [33], as well as entropy features for the Driver Fatigue data [33], with results shown in Figure 6. This is done to showcase that inter-participant variability is present across many tasks and participant populations and demonstrates it visually to complement the quantitative empirical results within Section 4. For each of the graphs in Figure 6, we see that the majority of the data are clustered by participant, meaning that most of the participant data belong to its own unique input distribution, with some overlap and similarity between participants. However, there are some limitations of t‑SNE that are worth noting and that are not obvious, and without their understanding, they can lead to incorrect assumptions about the underlying structure of the data. One limitation is that the cluster sizes in a t-SNE plot do not relate to distance between points of the cluster, as the algorithm adapts “distance” to each of the local clusters in the dataset, meaning dense clusters are expanded and sparse clusters are contracted [17]. This means that the sparsity of the cluster cannot be implied to have meaning. Another limitation is that the global geometry of the plot is not reliable as a source of information, meaning that the distances between clusters may or may not be accurate methods of interpreting the high-dimensional data in 2D space. While it is possible to dial in the hyperparameters to the correct values so that the 2D space does accurately represent the global geometry of the data in high-dimensional space, this requires a priori knowledge of the underlying structure of the high-dimensional data, which is unavailable. The implication of these limitations is that when interpreting t-SNE plots, the focus should be on simply the number of clusters present in the data and how they relate to the training dataset and the testing dataset. Any other information within the plot should not be taken as evidence of the underlying structure of the data in high-dimensional space. These limitations are important in understanding the data presented in the next section.

### 3.3. Reducing Inter-Participant Variability

As mentioned in Section 2.2, current approaches to EEG modeling are classified as either within-participant or cross-participant. Due to inter-participant variability, cross-participant models tend to always have lower classification accuracies than within-participant models, despite the fact that more participants typically also result in a larger training dataset for the model. 

In order to demonstrate these effects of inter-participant variability within cross-participant models, we study the phenomenon with synthetically altered data through transformation. To generate the data, we utilize two mutually exclusive, independent applied data transformations named *shifted Heaviside* (our own naming for the transformation for the purpose of discussion) and *shift to median*. The goal of these transformations is to reduce the inter-participant variability of the data while still preserving the local structure of each participant’s EEG data. In this manner, it can then be seen that as inter-participant variability is reduced and participants become more similar and no longer have different input distributions, classification performance improves because the effect of the covariate shift has been reduced. The purpose of this exploration is to demonstrate this performance-affecting relationship of inter-participant variability and covariate shift; we do not recommend utilizing these transformations in practice for the purpose of improving model performance.

The apparent performance improvement that occurs when data are transformed to reduce inter-participant variability implies that there will likely be overestimated classification performance in cross-participant models that are improperly validated and tested. When a model uses the same participants for both training and validation or testing, the higher measured performance is due to the reduced inter-participant variability between the training dataset and the validation or testing dataset—essentially masking the true differences that would exist between the people the model was trained on and the people the model was intended to be used on in the future. Similarly, when we apply transformations to reduce inter-participant variability, the goal is to transform the data in a manner such that multiple participants appear as if they belong to a single participant, and we can induce the effect of masking the true differences.

The transformation *shifted Heaviside* is both participant-based and feature-based. As mentioned at the beginning of this section, the name *shifted Heaviside* is the name we use in this paper to refer to this transformation proposed by Arevalillo-Herraez et al. in [36], based on the Heaviside function, as this transformation was not named by its originators. It was proposed by Arevalillo-Herraez et al. specifically for the use of reducing inter-participant variability in EEG data, and it does so by using the median value for each feature of each participant in order to map the original feature vector into a binary feature vector of the same size [36]. The effect of this transformation can be thought of as having the effect of shifting the data to the different corners of a hypercube. To create the mapping, first, the median value of each feature of each participant is calculated. Then, the original feature vector data are converted to a binary encoded vector where each feature value is transformed to a 1 if the value is greater than the median of the feature vector, or a 0 if less than or equal to the median (akin to a shifted Heaviside function). Specifically, they formulate their algorithm as follows: for *p*th participant, for all feature vectors $x_{p,j,}\;j=1,2,\ldots ,n_{p}$ in the set of training samples $X_{p}$, compute the median vector $x_{p}^{\prime }$. Then, transform all feature vectors ***u*** for the same participant *p* according to Equation (5), where [*k*] denotes the *k*th element (feature) of the corresponding vector.

(5) $$\bar{u}\left[k\right]=\left\{\begin{array}{ll} 1 & u\left[k\right]>x_{p}^{\prime }\left[k\right],\\ 0 & u\left[k\right]\leq \;x_{p}^{\prime }\left[k\right], \end{array}\right.$$

The *shift to median* transformation involves calculating a center point for each output class *y* across all participants in feature space and then shifting by class *y* each participant’s data closer to those class center points so that each participant’s data distribution moves closer together (toward the calculated class centers), while still preserving differences within each participant’s individual data observations. The goal is to reduce inter-participant variability by shifting all participants to a similar range in feature space, while still preserving local structure within each participant, including class effect. The effect of this transformation can be thought of as shifting each participant’s entire cluster of data by a certain amount so that it is re-centered on a new point (performed by class *y*). Using the same symbols in the previous paragraph, we have the following algorithm.

*Shift to Median*—Variables are defined as follows: *y* represents class, *j* represents the observation, *p* represents the participant, and *N* represents the total number of training samples.

1. ∀y Calculate median vector ${\tilde{C}}_{y}$ across all feature vectors $x_{p,y,j}$ of all participants *p* = 1, … , P
  a. ${\tilde{C}}_{y}=\left\{\begin{array}{c} x_{y,\frac{N+1}{2}\;}\;N\;\mathrm{odd}\\ \frac{1}{2}\left(x_{y,\frac{N}{2}}+x_{y,\frac{N+1}{2}}\right)\;N\;even \end{array}\right.$
2. $\forall p\;\forall y\;\forall x_{p,j}$ Calculate median centroid ${\tilde{c}}_{p,y}$ of *p*
  b. ${\tilde{c}}_{p,y}=\left\{\begin{array}{c} x_{p,y,\frac{N+1}{2}\;}\;N\;\mathrm{odd}\\ \frac{1}{2}\left(x_{p,y,\frac{N}{2}}+x_{p,y,\frac{N+1}{2}}\right)\;N\;even \end{array}\right.$
3. $\forall y\;\forall x_{p,j}$ Compute shifted vector $x_{p,y,j}^{\prime }$ = $x_{p,y,j}$ + $\left({\tilde{C}}_{y}-{\tilde{c}}_{p,y}\right)$

This results in three different datasets: original dataset, *shifted Heaviside* transformation, and *shift to median* transformation. Employing t-SNE on the datasets allows us to view the local clusters within the data. For EEG specifically, this typically allows us to identify clustering by participant, showcasing the inter-participant variability inherent across participants. To demonstrate this clustering as well as the EEG data transformations described above, we utilize the Driver Fatigue dataset [33] described in Section 4.1. 

This dataset contains both entropy and spectral features. In information theory, the entropy of a time series quantifies its regularity and predictability over time [37], and the entropy features extracted for use include approximate entropy (AE), sample entropy (SE), and fuzzy entropy (FE) features [38]. The spectral features were extracted using Morlet wavelet transforms in MATLAB to determine the frequency-domain mean power of two of the five clinical frequency EEG bands: alpha (12–15 Hz) and beta (16–22 Hz) ([5] pp. 151–174). Two frequency-spectral-power features extracted from EEG were computed for each of the 30 channels. This results in 60 features for the spectral feature space and 90 features for the entropy feature space (three entropy measures across all 30 channels).

**Figure 7:**
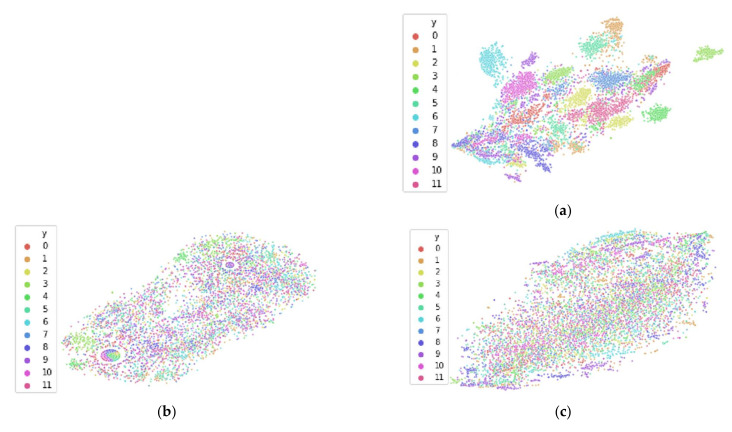
Results of visualizing the data using t-SNE for the entropy feature space before and after various data transformations: (**a**) Before any transformations; (**b**) After applying *shifted Heaviside* transformation; (**c**) After applying *shift to median* transformation. Colors correspond to different participants, with the same color applied to the same participant in each figure. Note in (**b**,**c**) that there is a lack of local clustering, implying that inter-participant variability has been reduced due to the transformations. The dimensions of t-SNE are arbitrary distances which represent that closer neighboring points in low-dimensional space are likely to be neighbors in high-dimensional space.

**Figure 8:**
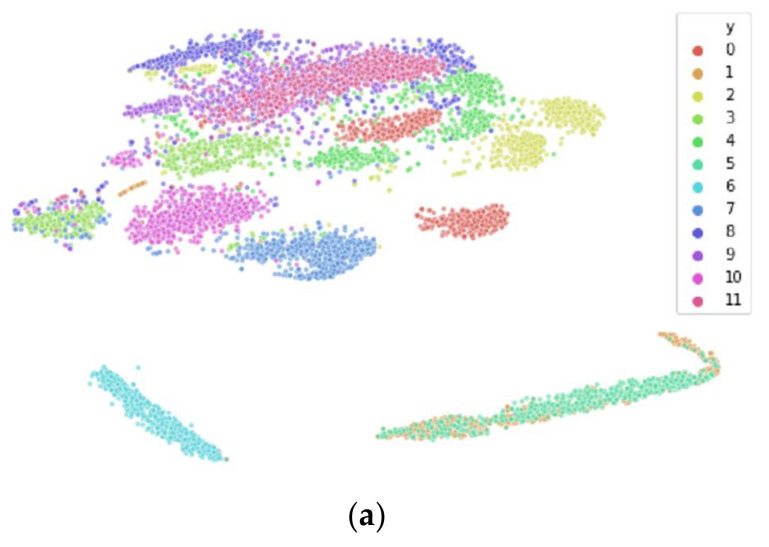

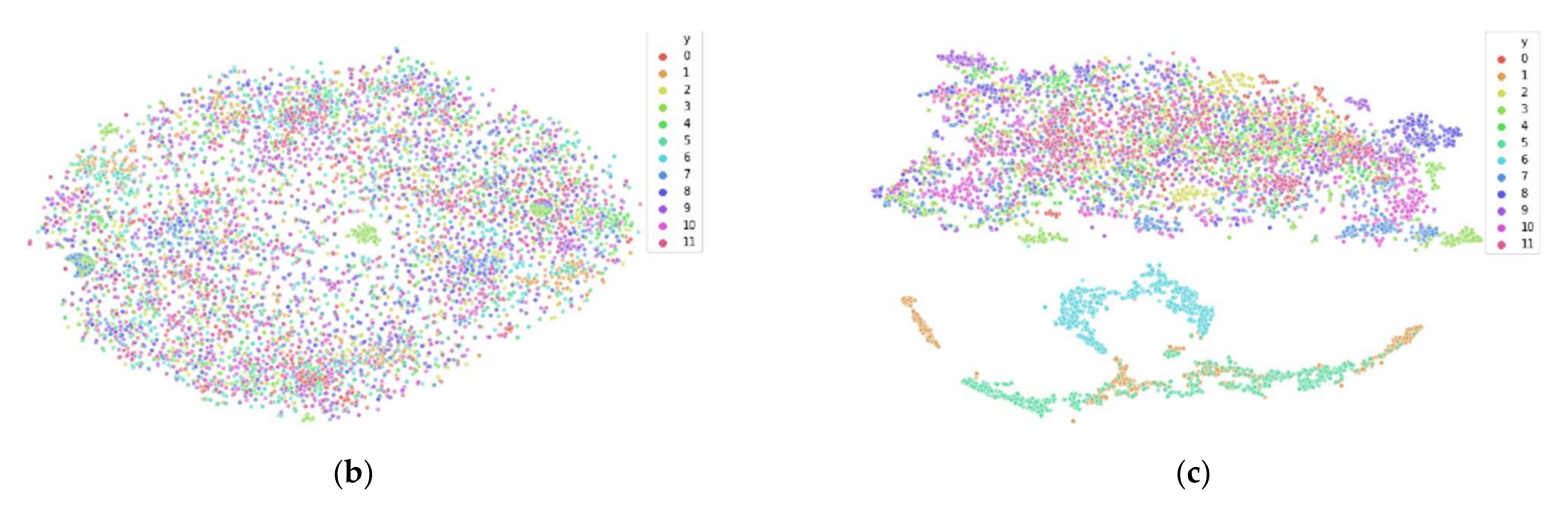
Results of visualizing the data using t-SNE for the spectral feature space before and after various data transformations: (**a**) Before any transformations; (**b**) After applying *shifted Heaviside* transformation; (**c**) After applying *shift to median* transformation. Colors correspond to different participants, with the same color applied to the same participant in each figure. The dimensions of t-SNE are arbitrary distances that represent that closer neighboring points in low-dimensional space are likely to be neighbors in high-dimensional space.

Figure 7a and Figure 8a both illustrate the results of applying t-SNE to the untransformed Drive Fatigue datasets for the entropy and spectral feature spaces, respectively. It can be seen that in these high-dimensional data spaces of 90 and 60 features each that there is significant clustering by participant, with coloring corresponding to a participant’s data. Note that this coloring has no effect on the t-SNE algorithm itself and is applied afterwards for visualization. As mentioned earlier in Section 3.2, due to the limitations of t-SNE, we cannot reliably interpret any information from the 2D plot outside of the number of clusters. Clusters found within t-SNE should only be treated as such: that they are localized clusters that exist within the high-dimensional data. After a data transformation, if t-SNE is unable to find local clustering despite hyperparameter tuning, then local clustering does not exist [17]. For these datasets, a lack of local clustering means that the inter-participant variability has been reduced to the point that t-SNE can no longer distinguish between participants in the feature space.

Figure 7b,c reveal the different data transformation’s effects on local clustering within the entropy feature spaces and Figure 8b,c show the transformation’s effects on the spectral feature spaces. For the entropy feature space, we see that each transformation has reduced the inter-participant variability to the point where t-SNE no longer finds local clustering within the data. Similarly, for the spectral feature space, we see that the *shifted Heaviside* transformation has the same result, while the *shift to median* transformation largely reduces local clustering within t-SNE, but not to the same effect as the *shifted Heaviside* transformation.

To demonstrate the effects of reducing inter-participant variability on classification accuracy in cross-participant models, cross-participant models were also built using each of these three datasets of data within both of the feature spaces (entropy and spectral). As this is the Driver Fatigue dataset, models were trained according to the methodology specified in Section 4.1. For each of the three subsets of data within both of the feature spaces of entropy and spectral features, separate models were trained and tested according to both the improper and proper methods of cross-participant model generation. For proper model generation, we follow the guidelines specified in Section 2.3, resulting in 12-fold LOPO CV. As mentioned in Section 4.1, for improper model generation, in order to match the number of folds (and data per fold) in LOPO CV, 12-fold CV was used with all participant data shuffled together and split across 12-folds. Together, this results in 12 models generated for each method.

Table 1 contains the classification accuracy results for each of the 12 models. It can be seen that for both the entropy and spectral feature spaces that improper model testing did not benefit from the data transformations. Intuitively, this makes sense, as these models are tested improperly; thus, the model has seen each participant’s input distribution, and therefore, a reduction of inter-participant variability is not impactful to the model. However, for proper model testing, we see that for both the entropy and spectral feature spaces that the *shift to median* transformation results in a dominance in accuracy of the 95% confidence interval (CI) from the transformation in comparison of the 95% CI’s. While the *shifted Heaviside* transformation did result in a reduction of inter-participant variability for both feature spaces as shown in Figure 7b and Figure 8b, this reduced inter-participant variability did not result in any significant effects on cross-participant model performance, suggesting that this transformation may be best suited for only certain datasets which its developers Arevalillo-Herraez et al. work with.

## 4. Empirical Demonstrations in Diverse EEG Case Studies

In this section, we utilize five publicly available datasets to empirically demonstrate the difference in machine learning performance results of using proper versus improper methods of dataset partitioning during training, validation and testing. These five datasets were selected to encompass diversity across the research activities using machine learning and EEG, to demonstrate the importance of following the proper methodology in many situations. The domains of the five datasets differ substantially in both tasks performed during data collection and subsequent classification using EEG, including both classification of different mental states within an individual: mental fatigue (*Driver Fatigue*), emotions (*Confused Students*), as well as determining of the existence of longer-term chronic conditions in individuals: mental disease (*Alcoholism)*, psychological conditions (*PTSD*), and mental disorders (*Schizophrenia*). In the chronic condition datasets, each participant (and all of the observations corresponding to that participant) are either in the chronic condition class or the class representing normal. Summary details of these datasets can be seen in Table 2. 

Model architectures used are selected based on research papers with top performance in their respective dataset and/or domain, with replication performed as closely as possible. In some cases, research papers were missing details about hyperparameters and other model details, and these details had to be selected using best practices of machine learning. With architecture and hyperparameters selected, two models are then created and evaluated separately using the same architecture and hyperparameter sweep (grid search utilized):

- Improper: trained, tuned, and evaluated during tests using all participant data.
- Proper: trained and tuned using data from a subset of the participants, then, during the test, evaluated using only data from participant(s) that were not used to train or tune the model.

Then, results of the two methods are contrasted and compared, with error rates displayed in a summary table in Section 5. It is also worth noting that the amount of data used for training and validation/testing is kept consistent across both the proper and improper methods, meaning that both models have the same quantity of observations to train upon, and additionally, both models are validated and tested with the same number of observations. This ensures that there is minimal difference between the two models in terms of architecture, hyperparameter sweeps, or the amount of data used for training, validation, or testing, and that the only difference between the models is the restriction surrounding which participants are used for training, validation, and testing for the proper method vs. the improper method. 

The next five subsections are structured as case studies for each of the five datasets, and they are in the following order: *Driver Fatigue*, *Confused Students*, *Alcoholism*, *PTSD*, and *Schizophrenia*. Each case study first discusses the purpose of the experiment, how it was conducted, and what EEG data were collected (pre-processing details are provided in Appendix A). Then, information on the model architecture and its methodology are provided, as well as the results previous researchers had achieved using that methodology. Then, we detail our own methodology to include having to fill any gaps missing from their architecture or hyperparameter selection, as well as how we perform both improper and proper training, validation, and testing for the two different models. Finally, we state results achieved with both models and compare them.

### 4.1. Driver Fatigue

This dataset is available on Figshare [33] through a link provided in Min et al.’s paper, which details both the experiment and the subsequent deep learning performed [38]. Their experiment consisted of collecting EEG recordings during a driving simulator for the purpose of using these signals to develop a model that could detect driver fatigue using EEG signals. Twelve participants used the driving simulator for 1–2 h in a highway setting with low traffic density, with EEG recorded in two phases during the session. The first phase consisted of 20 min of continuous driving, with the last 5 min of this 20-min segment recorded and labeled as the *normal* state. The second phase consisted of driving that lasted for 40–100 min until the participant’s self-reported questionnaire indicated that they were fatigued (surveys used were Lee’s Subjective Fatigue Scale [39] and the Chalder Fatigue Scale [40]), in which the last 5 min of driving were recorded in the EEG and labeled as the *fatigue* state. EEG was recorded using a 32-channel electrode cap, with two of the channels being reference channels linked to mastoid electrodes. The 5 min of EEG from each phase were epoched into 1 s segments for 300 epochs per phase per participant, resulting in a total of 3600 trials for the normal state and 3600 trials for the fatigue state. Then, the data were randomly split into training and testing datasets at a 50/50 ratio, without participants taken into account, thus resulting in improperly created datasets for cross-participant models. Feature extraction included several entropy measures, which were extracted for each trial and then normalized. In information theory, the entropy of a time series quantifies its regularity and predictability over time [37], with the measures extracted including approximate entropy, sample entropy, and fuzzy entropy [38].

In Min et al.’s work, these entropy features were then utilized for multiple classifiers, with the classifier that achieved the highest accuracy being an artificial neural network (ANN) [38]. The ANN had three layers, each with 20 hidden units and sigmoid activation functions. Gradient descent was used with mean squared error (MSE) for the loss the function, and the Levenberg–Marquardt function was used as the optimization function [41]. Leave-One-Out Cross-Validation (LOOCV) was utilized to report test classification accuracy, with their reported test accuracy being 0.968 or an error rate of 0.032.

The architecture above was followed for training both of our models; however, 12-fold CV was utilized, as there are 12 participants and Leave-One-Participant-Out (LOPO) CV results in 12-fold CV. Thus, for improper training and validation, 12-fold CV was used with all participant data shuffled together and split across 12-folds, and for proper training and validation, LOPO CV was used. Using this configuration, for improper testing of the cross-participant model, the best accuracy we obtained was 0.83, which was much lower than Min et al.’s reported test accuracy of 0.968 with their 50/50 training/testing split. In an effort to improve upon this, a hyperparameter sweep was conducted across hidden units (20, 30, 40, and 50), dropout rate (0.0, 0.1, 0.2), different learning rates (0.01, 0.001, 0.0001), and the *reduce_lr* callback of reducing the learning rate based on the number of epochs trained. The configuration with the highest classification accuracy for the improper method was one of 50 hidden units, 0.2 dropout rate, 0.001 learning rate, and *reduce_lr* callback was utilized. This hyperparameter sweep was also conducted for the proper method, with the configuration with the highest classification accuracy for the proper method being 40 hidden units, 0.2 dropout rate, 0.001 learning rate, and *reduce_lr* callback being utilized. Then, these configurations were used for improper and proper training and validation of the cross-participant models, respectively.

For improper training and validation of the cross-participant model using our configuration above, the reported classification accuracy using 12-fold CV was 0.91 (95% CI: 0.903, 0.917) or an error rate of 0.09 (95% CI: 0.083, 0.097). While this result is significantly lower than Min et al.’s error rate (0.09 vs. 0.032 [38]), our accuracy is still similar enough in magnitude for our goal of contrasting proper and improper methods of model evaluation. As such, when we built the model properly and trained and validated it using LOPOCV, the resulting accuracy was 0.540 (95% CI: 0.528, 0.552) or an error rate of 0.46 (95% CI: 0.448, 0.472). This error rate is over five times as that of the error rate of the improper method, illustrating how difficult classification of unseen participants is, and how significantly overestimated test accuracies can become by following an improper methodology, which does not account for the significance of inter-participant variability.

### 4.2. Confused Students

Participant data for this dataset are available on Kaggle [12] and come from an experiment involving college students. The purpose of the experiment was to collect EEG from college students while they were in a *confused* state and a *not confused* state and then build a model that could determine if the student was *confused* or *not confused* using the EEG signals. Researchers collected EEG while the students watched online education videos in a *confused* state and a *not confused* state [42]. Ten young adult college students watched two-minute online education videos (lectures) on various topics, which were assumed to not confuse an average college student, such as basic algebra and geometry, as well as topics that would be confusing, such as quantum mechanics and stem cell research. Each student watched five randomly selected videos from each category, and after each video, students self-rated their confusion on a scale of 1 (least confused) to 7 (most confused). EEG was recorded at a sampling rate of 512 Hz using a single-channel NeuroSky MindSet device, which has a single electrode that rests over the middle of the forehead, and two electrodes for ground and reference, each in contact with an ear. The first 30 s and last 30 s of each session’s EEG recording were removed in case the student was not ready; the middle 60 s was available for analysis. Then, NeuroSky software was used to extract features from the signal at 2 Hz to include the mean of the raw signal, mean power for the five traditional frequency bands (to include alpha low/high, beta low/high, and gamma low/high), and MindSet’s proprietary “attention” and “meditation” signals.

In addition to the experiment data, Kaggle also lists references with some of the latest classification results to use these data for the purpose of binary classification of whether a student is *confused* or *not confused*. The two references with the greatest classification accuracies both use bidirectional Long Short-Term Memory (LSTM) models as their neural network architecture [43,44], with the one we selected for replication being work from Ni et al., as their work provided the most detail for replication [44]. For Ni et al.’s work, each session consisted of a single trial as to provide sequence data for the recurrent neural network (RNN). Sessions from all nine participants were merged together for a cross-participant model, and 5-fold cross-validation was used across all participants (improper method). EEG features used consisted of proprietary measures from the MindSet EEG device labeled *Attention* (measure of mental focus) and *Meditation* (measure of calmness), the raw EEG signal values, and mean values of eight different frequency regions in the power spectrum. In addition to EEG signals, Ni et al. also opted to use the “Predefined Label” of whether a session was confusing or not as a feature. The bidirectional LSTM had 50 hidden units and used a tanh activation function, and it was followed by a fully connected layer with a sigmoid activation function. Before the bidirectional LSTM, batch normalization was used. No other architecture or hyperparameter methodology was provided. The CV test accuracy varied between 0.71 and 0.74 for their work, with an average 5-fold CV accuracy of 0.733.

To reproduce Ni et al.’s results for the improper model, the architecture above was followed along with the hyperparameters provided, and all of the EEG features were utilized, resulting in 11 total features used for training (in our replication of the research, the non-EEG “Predefined Label” feature was omitted; we do not recommend including a class label as a feature per standard practices of machine learning). In an effort to replicate their methodology of hyperparameter selection for the proper model, a hyperparameter grid search was performed across hidden units (40, 50, 60), dropout rate (0.0, 0.1, 0.2), and learning rate (0.001, 0.0001), with the highest performing proper model having hyperparameters of 50 hidden units, 0.0 dropout rate, and 0.0001 learning rate; the same hyperparameters Ni et al. and our improper model design used. This hyperparameter sweep ensures both the improper and proper models have selected their best hyperparameters for their input data. In an effort to increase the amount of training samples for the models, the EEG data were also segmented using a sliding sequence window of 15 samples in length and slides by 12 samples. Then, we built two separate cross-participant models using improper training and testing for one and proper training and testing for the other. The improper model utilized 5-fold CV for training and validation with all participant data shuffled together, resulting in every fold including some data from every participant (exact same method used by Ni et al.). The proper model also utilized 5-fold CV; however, it was Leave-Two-Participants-Out CV. Outside of this change in how the folds were formed for CV, all other variables remained the same between the models, to include architecture, features used, hyperparameters, and the number of observations used for both training and validation.

For improper training and validation of the cross-participant model using 5-fold CV, our replication of Ni et al.’s configuration [44] resulted in a test accuracy of 0.69 (95% CI: 0.654, 0.726), which was close to their reported test accuracy of 0.733, with a difference in error rates of 0.31 (95% CI: 0.274, 0.346) vs. 0.267. However, our proper training and validation of the proper cross-participant model using Leave-Two-Participants-Out CV resulted in an accuracy of 0.584 (95% CI: 0.552, 0.628) or an error rate of 0.416 (95% CI: 0.372, 0.448). The error rate of the proper method is over 33% greater than the error rate of the improper method, suggesting that improper training and testing of EEG data can lead to overestimation of model performance on unseen participants and thus the human population in general. 

### 4.3. Alcoholism

Participant data for this dataset are available from both Kaggle [12] and the University of California, Irvine (UCI) machine learning data repository [13], with this research utilizing the *Full Dataset* from the UCI repository. The source of the data comes from one of a number of experiments sponsored by the National Institute on Alcohol Abuse and Alcoholism (NIAAA) in the early 1990s, which were conducted with the purpose of recording brain activity during a task that was expected to elicit differences in the neural activity of healthy participants and alcoholic participants [45,46]. In the control group, there were 45 male participants, and in the experimental group, there were 77 alcoholic male participants. The task used was a visual object recognition task: the participant was presented with a sequence of two images and had to determine whether the second image was the same as the first. Signals were recorded from 64 scalp EEG electrodes and 2 electrooculography (EOG) electrodes, at a sampling rate of 256 Hz, and were referenced to node site Cz during EEG measurement. This resulted in a sequence dataset with 64 features × 256 µV values for each of the (approximately) 100 observations per participant.

Recently, the *Full Dataset* from the UCI repository was utilized by Farsi et al. to train both ANN and LSTM classifiers, with their LSTM architecture having the best performance with a reported test accuracy of 0.93 [47]. They used improper dataset partitioning, mixing the participants data and selecting 80% of the data for training and 20% for testing. For improper training and validation of the cross-participant model, we used 5-fold CV to align with Farsi et al.’s 80% training 20% testing dataset preparation. For proper cross-participant model evaluation, we utilized 5-fold Leave-N-Participants-Out CV, with N equal to 24 or 25 depending on the fold. Although the paper provided an architecture, it did not explicitly identify their choice of best hyperparameters that were selected for their final LSTM model—they only provided a list of what hyperparameters were explored. Therefore, in an effort to recreate their work, we utilized the architecture they specified and performed a hyperparameter sweep across all of the hyperparameters that were explored by the authors. This resulted in a 3-layer LSTM with layers and hidden units as follows (100-(Dropout Layer)-32-1), and a hyperparameter sweep performed for activation function (Relu, tanh, Sigmoid), dropout rate (0.2, 0.4), optimizer (Adam, SGD), batch size (50, 150), learning rate (0.1, 0.0001), epochs (50, 100), and loss function (MSE, Binary Cross Entropy). The resulting models from these hyperparameter sweeps performed poorly for both improper and proper models, so we instead used a 3-layer LSTM architecture with descending hidden units (H) across the three layers (H, H-50, H-100), dropout and recurrent dropout activated for all three layers, with activation function tanh, recurrent activation function sigmoid, batch size 256, optimizer Adam, learning rate 0.0001, and loss function Binary Cross Entropy. Then, we performed a hyperparameter sweep for this architecture across hidden units (200, 250, 300, 350), dropout rate (0.2, 0.3, 0.4), and epochs (200, 300, 400, 500). This architecture and its hyperparameter sweep had better performance, so we opted to use it as our final architecture for both the improper and proper methods of model creation. The best configuration for the improper model had hyperparameters of hidden units 350, dropout rate 0.4, and epochs 500. The best configuration for the proper model had hyperparameters hidden units 300, dropout rate 0.4, and epochs 400.

The resulting improper model had a test accuracy of 0.84 (95% CI: 0.82, 0.86) or an error rate of 0.16 (95% CI: 0.12, 0.18). While this result is significantly lower than Farsi et al.’s error rate (0.16 vs. 0.07 [47]), our accuracy is still similar enough in magnitude for our goal of contrasting proper and improper methods of model evaluation. The resulting proper model had a test accuracy of 0.69 (95% CI: 0.67, 0.71) or an error rate of 0.31 (95% CI: 0.29, 0.33), which is close to chance accuracy of 0.64 or a chance error rate of 0.36, as this dataset was imbalanced with a majority class of alcoholics. The error rate of the properly data-partitioned model is almost twice as large as the error rate of the improper model, again suggesting that if the goal is to build a model that can be used to make accurate estimates on unseen individuals, then the EEG cross-participant model must be evaluated properly by evaluating it only using data from participants not used during training or validating the model. 

### 4.4. Post-Traumatic Stress Disorder (PTSD)

This publicly available PTSD dataset can be found on Figshare [34] through an appendix and link provided in Rahmani et al.’s paper, which details the experiment used and their subsequent EEG analysis [34] (unrelated to machine learning). Researchers captured resting-state EEG from six healthy control (HC) participants and six combat-related PTSD participants, while they had an MRI taken, with the goal being to find differences between HCs and PTSD participants through analysis of the EEG. For this dataset, there were 33 channels of EEG recorded, with two of the 33 channels being used for ground and reference, and at a sampling rate of 5000 Hz. EEG preprocessing was performed in both the proprietary software BrainVision Analyzer2 and within EEGLAB. ICA was used to remove blink and saccade artifacts, and time periods containing motion artifacts from observed participant head motion were also removed. After artifact removal, the EEG was down‑sampled to 250 Hz. Scans lasted 526 s, and the first 6 s were removed for steady-state signals, resulting in 520 s of raw voltage value data per participant. However, only the first continuous 50,000 data points without participant motion were used within Rahmani et al.’s analysis, and this was subsequently the case with the data uploaded and made available to the public, resulting in 200 s of raw EEG per participant being available for machine learning. Then, EEG signals were segmented into 1-s non-overlapping epochs, resulting in 200 observations per participant. For feature selection, spectral features were extracted for the 31 EEG channels using Morlet wavelet transforms in MATLAB to determine the frequency-domain mean power of the five traditional frequency bands: delta (2–4 Hz), theta (4–8 Hz), alpha (8–12 Hz), beta (15–30 Hz), and gamma (30–80 Hz) [5] pp. 151–174. The mean power of these five bands for all 31 channels results in a total of 155 features (31 × 5) for each of the 260 observations for each of the 12 participants.

Since this dataset has not yet been used for published research in the area of machine learning, there is no machine learning workflow we are attempting to replicate; instead, we utilize a standard fully connected multi-layer perceptron neural network (MLPNN) for our architecture, which is a common and most fundamental ANN.

A hyperparameter sweep was performed to find a good model. The sweep was conducted across the following hyperparameters: hidden layers (1, 2), hidden units (20, 30, 40, 50), dropout rate (0.0, 0.1, 0.2), and learning rates (0.01, 0.001, 0.0001) for both the improper and proper methods of model evaluation, and the hyperparameter configuration that resulted in the highest validation accuracy was selected for each method. The architecture used ReLU activation functions for dense layers, a Sigmoid activation function for the output layer, and ‘Adam’ for the optimizer; training was conducted for 50 epochs. For training and validation of the improper model, 12-fold CV was used with all participant data shuffled together and split across the 12-folds, and for training and validation of the proper model, 12-fold LOPO CV was used.

The best configuration for the improper model consisted of 1 hidden layer, 50 hidden units, a learning rate of 0.001, and a dropout rate of 0.2. This configuration resulted in a 12-fold CV accuracy of 0.995 (95% CI: 0.9922, 0.9978) or an error rate of 0.005 (95% CI: 0.0022, 0.0078). The best configuration for the proper model was similar in that it consisted of the same parameters for everything except the hidden units being 40 instead of 50. This configuration resulted in a 12-fold LOPO CV of 0.803 (95% CI: 0.7871, 0.8189) or an error rate of 0.197 (95% CI: 0.1811, 0.2129). This results in an error rate that is over 39 times larger for the proper method versus the improper method of training and validation, which is the 2nd largest difference between proper and improper partitioning within these case studies. Relying on the overly optimistic, extremely low error rate measured in the performance of the model trained using the improper training method would falsely drive overconfidence in the model’s performance in future use. Once again, the evidence suggests that if the intent is to estimate performance on new people, proper segregation of participants in the partitioning of the training, validation, and test datasets is paramount.

### 4.5. Schizophrenia

This dataset is available on Kaggle [35] and was collected in an effort to study the difference in corollary discharge between participants with schizophrenia and those without schizophrenia (HCs) [48]. The participant’s task was to either (1) press a button every 1–2 s to deliver an 80 dB tone, (2) passively listen to that same tone, or (3) press a button that did not produce a tone or any other effect other than the tactile response of depressing the button. Each event condition occurred a total of 100 times for each participant, resulting in 300 trials per participant. In total, in the dataset there were 32 HCs and 49 patients with schizophrenia; however, data from only 40 participants were available online (25 HCs and 15 diagnosed with schizophrenia).

Data were collected using a BioSemi ActiveTwo 64 + 2 electrode cap, with 64 scalp sites and 2 references electrodes placed over the mastoids [48]. Data were sampled at 1024 Hz and epoched at 3 s for each trial, with the start of each epoch being time-locked to 1.5 s before button press. The EEG data were uploaded to Kaggle [35] in two different formats, one in time-series as raw EEG voltage values, and the other with event-related potential (ERP) features. In order to generate richer features for machine learning, spectral features were extracted from the raw EEG voltage values for all 64 channels. This was done similarly as done in the PTSD dataset, using Morlet wavelet transforms in MATLAB to determine the frequency-domain mean power of the five traditional frequency bands: delta (2–4 Hz), theta (4–8 Hz), alpha (8–12 Hz), beta (15–30 Hz), and gamma (30–80 Hz) ([5] pp. 151–174). This resulted in 320 features for each observation (64 channels × 5 frequency bands = 320), with participants having between 280 and 290 observations each. Unfortunately, the dataset was heavily imbalanced, with 25 participants being HCs, and only 15 participants being diagnosed with schizophrenia. To alleviate this imbalance, only 15 of the 25 HCs were randomly selected to be used for machine learning.

For our architecture selection for this dataset, we use both a neural network, as well as a more traditional machine learning model—the random forest classifier. Buettner et al. achieved high levels of accuracy for EEG classification of HCs vs. participants with schizophrenia using an RFC [49] (albeit on a different EEG dataset), so they are a proven model type for this domain, with the neural network architecture implemented for additional investigation. The spectral features generated were utilized for both the MLPNN and the RFC, and both architectures followed both the proper and improper methods of model evaluation, resulting in four separate models generated. For improper training and validation, 30-fold CV was utilized with all participant data shuffled together and split across the 30-folds, and for proper training and validation, 30-fold LOPO CV was used. As with the PTSD dataset, we did not have a published neural network methodology to replicate for this dataset.

For the MLPNN architecture, a hyperparameter sweep was conducted across the following hyperparameters: hidden layers (1, 2), hidden units (20, 30, 40, 50), dropout rate (0.0, 0.1, 0.2), and learning rates (0.01, 0.001, 0.0001). This hyperparameter sweep was conducted for both the improper and proper methods of model evaluation, and the hyperparameter configuration that resulted in the highest validation accuracy was selected for each method. Other parameters of the architecture include using the ReLU activation function for dense layers, a Sigmoid activation function for the output layer, and ‘Adam’ for the optimizer; the number of training epochs set to 50.

RFC hyperparameters selected for hyperparameter tuning included the maximum depth of the trees and the number of features to consider. The number of estimators (trees) was determined by incrementally increasing the number of estimators by 5 from a low value of 50 until validation accuracy no longer improved. For this, maximum depth was set to its default sklearn value of ‘None’ so that there was no limit to depth, and the maximum features set to its typical recommended amount of $m=\sqrt{p}$ where *p* equals the 320 features, and thus, $m=\sqrt{320}=18$ [50]. By incrementally increasing the number of estimators by 5 from 50 to 750 as described above, 110 was found to result in the best validation accuracy, and this amount was used for both proper and improper methods of model evaluation for the RFCs. From here, a hyperparameter sweep for the number of features and the maximum depth was conducted, utilizing values from 1 to 25 for each. These values were determined by going far above and below the typical recommended values for these parameters (e.g., the square root of features for the number of max_features *m*) [50]. This resulted in a hyperparameter sweep of $25^{2}=625$ models for both the improper and proper methods of model evaluation, resulting in 1250 models in total generated during hyperparameter search.

The best configuration for the MLPNN improper model consisted of 1 hidden layer, 50 hidden units, a learning rate of 0.001, and a dropout rate of 0.2. This configuration resulted in a 30-fold CV accuracy of 0.992 (95% CI: 0.990, 0.9939) or an error rate of 0.008 (95% CI: 0.0061, 0.01). For the proper MLPNN model, there was no significant difference between any of the configurations, and no model was able to perform better than random chance (50%), illustrating how severe the effect of covariate shift can be in EEG data, depending on the participants used. The best configuration for the RFC improper model was maximum features set to 15 and maximum depth set to 24, resulting in a 30-fold CV accuracy of 0.941 (95% CI: 0.936, 0.946) or an error rate of 0.059 (95% CI: 0.054, 0.064). For the proper RFC model, similar to the proper MLPNN model, there was no significant difference between any of the configurations, and no model was able to perform better than random chance (50%). This final case study showcases the most significant effect of covariate shift, resulting in models that are unable to perform better than random chance due to the significant inter-participant variability that exists between the participants. 

## 5. Discussion

Our empirical results show that improper dataset evaluation can lead to unrealistic and overestimated accuracies for general population EEG cross-participant models. Table 3 specifies the extent of these differences in error rates between improper and proper methods, ranging from a 35% increase in error rate for the *confused students* dataset, all the way up to a 3900% increase in error rate in the case of the *schizophrenia* dataset. As mentioned in Section 4, the diversity of these datasets and the methods used provide evidence that performance overestimation due to improper data partitioning is indeed a phenomenon of EEG that is not unique to any one subset of experiment, task, participant, or equipment used, nor is it merely an aspect of only certain EEG features or types of machine learning models. Instead, the risk of performance overestimation is an inherent phenomenon of individual differences in EEG that should always be considered when developing general population EEG cross-participant models.

Proper care with EEG data preparation has been a subject of recent exploration by Li et al. as well [51]. Li et al. demonstrated that due to EEG’s non-stationarity, proper guidelines for the design of the experiment much be followed in order obtain model results that are not overestimated, particularly in the block design of the experiment so that stimuli of different classes are intermixed. If not followed, models instead learn to classify through arbitrary temporal artifacts, giving the false appearance of high performance. Our findings are synergistic with Li’s: we demonstrate the necessity of partitioning the data properly when performing machine learning on collected data *after* the experiment is complete; due to individual differences, proper care with EEG data partitioning by participant yields more accurate estimates of model results on future data. Together, both Li et al.’s guidelines for the design of the experiment and our guidelines for proper post-experiment dataset partitioning should be followed in order to obtain results for EEG cross-participant models that are representative of the model’s performance on the general population.

In Section 3, we demonstrated how t-SNE can be used to visualize covariate shift between participants due to their inter-participant variability, and we also illustrated how the *shifted Heaviside* and the *shift to median* transformations could be utilized to reduce this inter-participant variability. Additionally, for the purpose of demonstrating the relationship between this inter-participant variability and covariate shift, we explored the effect of these transformations in improving cross-participant model accuracy for both improper and proper model creation across two different feature spaces (*entropy* and *spectral* features). As can be seen in Figure 7 b,c and Figure 8b,c, both transformations were successful in reducing inter-participant variability for both feature spaces; however, only the *shift to median* transformation resulted in a dominant increase in accuracy of the 95% confidence intervals for both feature spaces for proper model creation, with the *shifted Heaviside* transformation having no improvement in model accuracy. In contrast to the *shifted Heaviside* results, Arevalillo-Herraez et al. (the originators of the *shifted Heaviside* transformation) had improvement of model accuracy in three different datasets they utilized, all of which were affect recognition-based datasets with arousal and valence features [36]. In their research, they also followed proper dataset partitioning guidelines and utilized LOPO CV. This suggests that a transformation that results in a reduction or elimination of inter-participant variability does not necessarily imply an improvement in cross-participant model accuracy.

## 6. Conclusions

As mentioned in Section 2, five out of six EEG deep learning models in research today are cross-participant models, with only one out of those five models following some method of proper dataset partitioning to ensure the model was tested with unseen participants [3]. Our empirical results show that models that utilize improper dataset evaluation have overestimated and unrealistic accuracies for the general population, with the difference in error rates for improper versus proper dataset evaluation ranging from a 35% increase in error rate up to a 3900% increase in error rate. These empirical findings suggest that if this trend continues, the body of research for EEG cross-participant models will become diluted with research that claims overestimated and unrealistic performance metrics, both downplaying the true difficulty in creating a high-performing EEG cross-participant model, and also slowing scientific progress of researching methodologies, which results in cross-participant models that are truly high performing for the general population. Thus, it is absolutely critical that the body of research corrects this trend and follows the proper dataset partitioning guidelines described in this research. Specifically, it means that:

- Data from participants used for model training must not be used for model validation or testing.
- Participants that are utilized for validation must not be used for testing.

This ensures the model is tested with unseen participants and reflects its intended purpose.

These findings extend beyond individual researchers. In addition, it is also important that data contributors, and the owners and maintainers of dataset repositories (e.g., Kaggle [12] and the UCI machine learning data repository [13]) managing human data ensure these guidelines are followed as well. Specifically, for these repositories, we recommend that:

- Any EEG data that are made available for download should always have (de-identified) participant labels available so that users may properly partition the data themselves.
- If the data contributors or maintainers decide to pre-partition the data into separate training and test datasets (as is sometimes done for competitions of machine learning models), then proper dataset partitioning guidelines should be followed for preparing those training and test datasets before they are made available for download by the general public.

We also recommend that the repository include these guidelines of proper dataset partitioning with all hosted EEG datasets, as this would help spread the word in regard to proper dataset partitioning and inform users who are unaware of inter-participant variability and its effects.

Lastly, we strongly recommend that the “Neurotechnologies for Brain–Machine Interfacing” group of the Institute of Electrical and Electronics Engineers Standards Association (IEEE SA) consider and adopt these guidelines for all future proposals of standards. In this group’s most recent *Standards Roadmap* [52], stakeholders and experts across government, academia, and industry identified the existing gap in the standardization of performance assessment and benchmarking for BMI as a clear priority for standardization [53]. Specifically, the proposal should identify these guidelines as a minimal reporting requirement for performance evaluation of EEG cross-participant models, leading to standardization in reporting how the data are partitioned, identifying their limitations, and curbing performance claims accordingly.

## Figures and Tables

**Table 1:** Classification accuracies for the 12 models generated from transformed and non-transformed driver fatigue data. Improper models were generated with the improper method of cross-participant model generation utilizing 12-fold CV with all participant data shuffled together and split across 12 folds, and proper model generation utilized 12-fold LOPO CV. The purpose of this table is two-fold. One is to depict that improper model generation typically results in overestimated model accuracy as can be seen with increased accuracies for improper vs. proper. The other is to depict the results of the proper method on untransformed data versus the proper method on the two transformed datasets. **Bold** signifies dominance in accuracy of the 95% confidence interval from the transformation in comparison of the 95% confidence intervals.

	Entropy	Spectral
**Improper**		
Untransformed	0.91 (0.89, 0.93)	0.82 (0.79, 0.85)
*Shifted Heaviside*	0.72 (0.68, 0.76)	0.66 (0.62, 0.70)
*Shift to Median*	0.91 (0.89, 0.93)	0.82 (0.79, 0.85)
**Proper**		
Untransformed	0.50 (0.46, 0.54)	0.50 (0.46, 0.54)
*Shifted Heaviside*	0.50 (0.46, 0.54)	0.47 (0.43, 0.51)
***Shift to Median***	**0.80 (0.77, 0.83)**	**0.72 (0.68, 0.76)**

**Table 2:** Details for the publicly available datasets. All datasets are binary classification tasks, and all datasets are balanced except for the Alcoholism dataset. This gives chance accuracy for Alcoholism defined as 0.64 and 0.50 for all other datasets.

Dataset	Year Collected	Binary Classification Task	# of Participants
Driver Fatigue [33]	2017	Normal vs. Fatigue	12
Confused Students [12]	2013	Confused vs. Not Confused	10
Alcoholism [13]	1999	Alcoholic vs. Non-Alcoholic	122
PTSD [34]	2018	Pre-Treatment vs. Post-Treatment	12
Schizophrenia [35]	2014	Schizophrenia vs. Healthy Control	30

**Table 3:** Validation results for the five case studies (95% CI). All datasets are binary classification tasks, and all datasets are balanced except for the Alcoholism dataset. This gives chance error rate for Alcoholism defined as 0.36, and 0.50 for all other datasets. Results should be compared within datasets (left to right) between the improper and proper method. The proper method always reveals a significantly greater error rate than the improper method, suggesting the risks of overestimation of performance, which can result from using the improper method.

Dataset	Architecture Used	Error Rate– Improper Method	Error Rate– Proper Method
Driver Fatigue	MLPNN	0.09 (0.083, 0.097)	0.466 (0.448, 0.472)
Confused Students	Bi-LSTM	0.31 (0.274, 0.346)	0.416 (0.372, 0.448)
Alcoholism	LSTM	0.16 (0.12, 0.18)	0.31 (0.29, 0.33)
PTSD	MLPNN	0.005 (0.0022, 0.0078)	0.197 (0.1811, 0.2129)
Schizophrenia	MLPNN	0.008 (0.0061, 0.01)	0.50 (0.44, 0.56)
Schizophrenia	RFC	0.059 (0.054, 0.064)	0.50 (0.44, 0.56)

## Data Availability

Publicly available datasets were utilized in this study. These datasets can be found at the following URL’s. Driver Fatigue: https://figshare.com/articles/dataset/The_original_EEG_data_for_driver_fatigue_detection/5202739 (accessed on 4 May 2021). Confused Students: https://www.kaggle.com/wanghaohan/confused-eeg (accessed on 4 May 2021). Alcoholism: https://www.kaggle.com/nnair25/Alcoholics (accessed on 4 May 2021). PTSD: https://figshare.com/articles/dataset/Dynamical_Hurst_analysis_identifies_EEG_channel_differences_between_PTSD_and_healthy_controls/6737795 (accessed on 4 May 2021). Schizophrenia: https://www.kaggle.com/broach/button-tone-sz (accessed on 4 May 2021).

## Abbreviations

ANN	Artificial Neural Network
BMI	Brain–Machine Interface
CI	Confidence Interval
CV	Cross-Validation
DL	Deep Learning
DR	Dropout Rate
EEG	Electroencephalography
EOG	Electrooculography
GRU	Gated Recurrent Unit
HC	Healthy Control
HU	Hidden Unit
IEEE SA	Institute of Electrical and Electronics Engineers Standards Association
iid	Independent and Identically Distributed
KL	Kullback-Leibler
LOPO	Leave-One-Participant-Out
LOOCV	Leave-One-Out Cross-Validation
LR	Learning Rate
LSTM	Long Short-Term Memory
MLPNN	Multi-Layer Perceptron Neural Network
Opt	Optimizer
PCA	Principal Component Analysis
PTSD	Post-Traumatic Stress Disorder
ReLU	Rectified Linear Unit
RFC	Random Forest Classifier
RNNt-SNE	Recurrent Neural Networkt-Distributed Stochastic Neighbor Embedding
UCI	University of California, Irvine

## Appendix A

In this appendix, we include any pre-processing details that were provided by the originators of the datasets used within this research.

### Appendix A.1. Driver Fatigue Data

EEG was recorded using a 32-channel electrode cap, with two of the channels being reference channels linked to mastoid electrodes [38]. Scan 4.3 software of Neuroscan was used for preprocessing, with raw signals filtered by a 50 Hz notch filter and a 0.15 Hz to 45 Hz band pass filter in order to remove noise.

### Appendix A.2. Confused Students Data

No known preprocessing information was provided by the originators.

### Appendix A.3. Alcoholism Data

EEG correlates were sampled from 62 scalp electrodes and two EOG electrodes, at a sampling rate of 256 Hz [13]. Sampling started at 190 ms before onset of stimulus in order to record a pre-stimulus baseline, and EEG correlate durations provided in the dataset were 1 s in duration. Sensor values were provided in µV, resulting in a sequence of 256 temporally organized values for each EEG channel. Trials with excessive eye or body movements (>73.3 µV) were rejected online. Only artifact free EEG segments were used to include eye blink artifacts. EEG electrodes were referenced to node site Cz during EEG measurement.

### Appendix A.4. PTSD Data

For this dataset, there were 33 channels of EEG recorded, with two of the 33 channels being used for ground and reference, and at a sampling rate of 5000 Hz [34]. EEG preprocessing was performed in the proprietary software BrainVision Analyzer2. MRI gradient artifacts and cardio ballistic artifacts were removed using the template subtraction method. Then, the EEG was down-sampled to 250 Hz and filtered with a 40 Hz low-pass filter. Then, ICA was applied to remove residual cardioballistic artifacts as well as blink and saccade artifacts. Time periods of head motion were removed.

### Appendix A.5. Schizophrenia Data

Vertical EOG (VEOG) and Horizontal EOG (HEOG) were also collected for the purpose of capturing eye movement and blinks [35]. Due to the size of the raw EEG signals, preprocessing was already performed on the dataset prior to its upload for public use. This preprocessing included re-referencing to the averaged mastoid channels, applying a 0.1 Hz high-pass filter, interpolation of outlier channels, and rejection of outlier components and outlier trials due to EEG artifacts using the FASTER artifact rejection method [54].

## References

[1] Hefron R.G., Borghetti B.J., Christensen J.C., Kabban C.M.S. (2017). Deep long short-term memory structures model temporal dependencies improving cognitive workload estimation. Pattern Recognit. Lett..

[2] Hefron R., Borghetti B., Kabban C.S., Christensen J., Estepp J. (2018). Cross-participant EEG-based assessment of cognitive workload using multi-path convolutional recurrent neural networks. Sensors.

[3] Roy Y., Banville H., Albuquerque I., Gramfort A., Falk T.H., Faubert J. (2019). Deep learning-based electroencephalography analysis: A systematic review. J. Neural Eng..

[4] Kaplan A.Y., Fingelkurts A.A., Borisov S.V., Darkhovsky B.S. (2005). Nonstationary nature of the brain activity as revealed by EEG/MEG: Methodological, practical and conceptual challenges. Signal Process..

[5] Cohen M.X. (2019). Analyzing Neural Time Series Data. Analyzing Neural Time Series Data.

[6] Sugiyama M., Krauledat M., Müller K.R. (2007). Covariate shift adaptation by importance weighted cross validation. J. Mach. Learn. Res..

[7] Raza H. (2016). Adaptive Learning for Modelling Non-Stationarity in EEG-Based Brain-Computer Interfacing. Ph.D. Thesis.

[8] Raza H., Prasad G., Li Y. Dataset shift detection in non-stationary environments using EWMA charts. Proceedings of the 2013 IEEE International Conference on Systems, Man, and Cybernetics.

[9] Raza H., Prasad G., Li Y. (2013). EWMA based two-stage dataset shift-detection in non-stationary environments. IFIP Advances in Information and Communication Technology.

[10] Raza H., Prasad G., Li Y. Adaptive learning with covariate shift-detection for non-stationary environments. Proceedings of the 2014 14th UK Workshop on Computational Intelligence (UKCI).

[11] Sugiyama M., Kawanabe M. (2012). Machine Learning in Non-Stationary Environments: Introduction to Covariate Shift Adaptation.

[12] Begleiter H., Neurodynamics Laboratory State University of New York Health Center at Brooklyn. http://www.downstate.edu/hbnl/.

[13] Dua D., Graff C. UCI Machine Learning Repository. http://archive.ics.uci.edu/ml.

[14] Raza H., Samothrakis S. Bagging Adversarial Neural Networks for Domain Adaptation in Non-Stationary EEG. Proceedings of the 2019 International Joint Conference on Neural Networks (IJCNN).

[15] Géron A. (2019). Hands-On Machine Learning with Scikit-Learn, Keras, and TensorFlow: Concepts, Tools, and Techniques to Build Intelligent Systems.

[16] Van Der Maaten L., Hinton G. (2008). Visualizing data using t-SNE. J. Mach. Learn. Res..

[17] Wattenberg M., Viégas F., Johnson I. (2016). How to Use t-SNE Effectively.

[18] Xiao H., Rasul K., Vollgraf R. (2017). Fashion-MNIST: A Novel Image Dataset for Benchmarking Machine Learning Algorithms. arXiv.

[19] Birjandtalab J., Pouyan M.B., Nourani M. An unsupervised subject identification technique using EEG signals. Proceedings of the 2016 38th Annual International Conference of the IEEE Engineering in Medicine and Biology Society (EMBC).

[20] Rasoulzadeh V., Erkus E.C., Yogurt T.A., Ulusoy I., Zergeroğlu S.A. (2017). A comparative stationarity analysis of EEG signals. Ann. Oper. Res..

[21] Li Y., Kambara H., Koike Y., Sugiyama M. (2010). Application of covariate shift adaptation techniques in brain-computer interfaces. IEEE Trans. Biomed. Eng..

[22] Matthews G., Amelang M. (1993). Extraversion, arousal theory and performance: A study of individual differences in the eeg. Pers. Individ. Dif..

[23] Medrano P., Nyhus E., Smolen A., Curran T., Ross R.S. (2017). Individual differences in EEG correlates of recognition memory due to DAT polymorphisms. Brain Behav..

[24] Landolt H.-P. (2011). Genetic determination of sleep EEG profiles in healthy humans. Progress Brain Res..

[25] Smit D.J.A., Boomsma D.I., Schnack H.G., Hulshoff Pol H.E., De Geus E.J.C. (2012). Individual Differences in EEG Spectral Power Reflect Genetic Variance in Gray and White Matter Volumes. Twin Res. Hum. Genet..

[26] Muthukumaraswamy S.D., Edden R.A.E., Jones D.K., Swettenham J.B., Singh K.D. (2009). Resting GABA concentration predicts peak gamma frequency and fMRI amplitude in response to visual stimulation in humans. Proc. Natl. Acad. Sci. USA.

[27] Muthukumaraswamy S.D., Singh K.D. (2013). Visual gamma oscillations: The effects of stimulus type, visual field coverage and stimulus motion on MEG and EEG recordings. Neuroimage.

[28] Cohen M.X. (2011). Hippocampal-prefrontal connectivity predicts midfrontal oscillations and long-term memory performance. Curr. Biol..

[29] Raza H., Rathee D., Zhou S.M., Cecotti H., Prasad G. (2019). Covariate shift estimation based adaptive ensemble learning for handling non-stationarity in motor imagery related EEG-based brain-computer interface. Neurocomputing.

[30] Goodfellow I., Bengio Y., Courville A. (2016). Deep Learning.

[31] Bickel S. (2008). Learning under Differing Training and Test Distributions. Ph.D. Thesis.

[32] Shimodaira H. (2000). Improving predictive inference under covariate shift by weighting the log-likelihood function. J. Stat. Plan. Inference.

[33] Min J., Wang P., Hu J. The Original EEG Data for Driver Fatigue Detection. https://figshare.com/articles/dataset/The_original_EEG_data_for_driver_fatigue_detection/5202739.

[34] Rahmani B., Wong C.K., Norouzzadeh P., Bodurka J., McKinney B. (2018). Dynamical hurst analysis identifies eeg channel differences between ptsd and healthy controls. PLoS ONE.

[35] Roach B. EEG Data from Basic Sensory Task in Schizophrenia. https://www.kaggle.com/broach/button-tone-sz.

[36] Arevalillo-Herráez M., Cobos M., Roger S., García-Pineda M. (2019). Combining inter-subject modeling with a subject-based data transformation to improve affect recognition from EEG signals. Sensors.

[37] Donnelly P., Ellis R.S. (1987). Entropy, Large Deviations, and Statistical Mechanics. J. Am. Stat. Assoc..

[38] Min J., Wang P., Hu J. (2017). Driver fatigue detection through multiple entropy fusion analysis in an EEG-based system. PLoS ONE.

[39] Lee K.A., Hicks G., Nino-Murcia G. (1991). Validity and reliability of a scale to assess fatigue. Psychiatry Res..

[40] Chalder T., Berelowitz G., Pawlikowska T., Watts L., Wessely S., Wright D., Wallace E.P. (1993). Development of a fatigue scale. J. Psychosom. Res..

[41] Yu H., Wilamowski B.M. (2011). Levenberg-marquardt training. Ind. Electron. Handb..

[42] Wang H., Li Y., Hu X., Yang Y., Meng Z., Chang K.M. Using EEG to improve massive open online courses feedback interaction. Proceedings of the 16th Annual Conference on Artificial Intelligence in Education (AIED).

[43] Ni Z., Yuksel A.C., Ni X., Mandel M.I., Xie L. Confused or not confused?: Disentangling Brain activity from EEG data using Bidirectional LSTM Recurrent Neural Networks. Proceedings of the 8th ACM International Conference on Bioinformatics, Computational Biology, and Health Informatics.

[44] Wang H., Wu Z., Xing E.P. Removing confounding factors associated weights in deep neural networks improves the prediction accuracy for healthcare applications. Proceedings of the Pacific Symposium on Biocomputing.

[45] Ingber L. (1997). Statistical mechanics of neocortical interactions: Canonical momenta indicators of electroencephalography. Phys. Rev. E.

[46] Zhang X.L., Begleiter H., Porjesz B., Litke A. (1997). Electrophysiological Evidence of Memory Impairment in Alcoholic Patients. Biol. Psychiatry.

[47] Farsi L., Siuly S., Kabir E., Wang H. (2021). Classification of Alcoholic EEG Signals Using a Deep Learning Method. IEEE Sens. J..

[48] Ford J.M., Palzes V.A., Roach B.J., Mathalon D.H. (2014). Did i do that? Abnormal predictive processes in schizophrenia when button pressing to deliver a tone. Schizophr. Bull..

[49] Buettner R., Hirschmiller M., Schlosser K., Rossle M., Fernandes M., Timm I.J. High-performance exclusion of schizophrenia using a novel machine learning method on EEG data. Proceedings of the 2019 IEEE International Conference on E-health Networking, Application & Services (HealthCom).

[50] Casella G., Fienberg S., Olkin I. (2013). An Introduction to Statistical Learning.

[51] Li R., Johansen J.S., Ahmed H., Ilyevsky T.V., Wilbur R.B., Bharadwaj H.M., Siskind J.M. (2021). The Perils and Pitfalls of Block Design for EEG Classification Experiments. IEEE Trans. Pattern Anal. Mach. Intell..

[52] Standards Roadmap: Neurotechnologies for Brain-Machine Interfacing, IEEE Standards Association, Piscataway, NJ, USA, Industry Connections Report IC17-007. https://standards.ieee.org/content/dam/ieee-standards/standards/web/documents/presentations/ieee-neurotech-for-bmi-standards-roadmap.pdf.

[53] Chavarriaga R., Carey C., Contreras-Vidal J.L., Mckinney Z., Bianchi L. (2021). Standardization of Neurotechnology for Brain-Machine Interfacing: State of the Art and Recommendations. IEEE Open J. Eng. Med. Biol..

[54] Nolan H., Whelan R., Reilly R.B. (2010). FASTER: Fully Automated Statistical Thresholding for EEG artifact Rejection. J. Neurosci. Methods.

